# Transcriptomic analysis of immune response to bacterial lipopolysaccharide in zebra finch (*Taeniopygia guttata*)

**DOI:** 10.1186/s12864-019-6016-3

**Published:** 2019-08-14

**Authors:** Cassandra S. Scalf, Julia H. Chariker, Eric C. Rouchka, Noah T. Ashley

**Affiliations:** 1 Department of Biology, Western Kentucky University, Bowling Green, KY 42101-1800 USA; 2 Department of Neuroscience Training, University of Louisville, Louisville, KY 40292 USA; 3 Department of Computer Engineering and Computer Science, University of Louisville, Louisville, KY 40292 USA

**Keywords:** Bird, Disease, Ecoimmunology, Hypothalamus, Immune response, Lipopolysaccharide, Red blood cells, RNA-seq, Spleen, Transcriptome, Zebra finch

## Abstract

**Background:**

Despite the convergence of rapid technological advances in genomics and the maturing field of ecoimmunology, our understanding of the genes that regulate immunity in wild populations is still nascent. Previous work to assess immune function has relied upon relatively crude measures of immunocompetence. However, with next-generation RNA-sequencing, it is now possible to create a profile of gene expression in response to an immune challenge. In this study, captive zebra finch (*Taeniopygia guttata*; adult males) were challenged with bacterial lipopolysaccharide (LPS) or vehicle to stimulate the innate immune system. 2 hours after injection, birds were euthanized and hypothalami, spleen, and red blood cells (RBCs) were collected. Taking advantage of the fully sequenced genome of zebra finch, total RNA was isolated, sequenced, and partially annotated in these tissue/cells.

**Results:**

In hypothalamus, there were 707 significantly upregulated transcripts, as well as 564 and 144 in the spleen and RBCs, respectively, relative to controls. Also, 155 transcripts in the hypothalamus, 606 in the spleen, and 61 in the RBCs were significantly downregulated. More specifically, a number of immunity-related transcripts (e.g., IL-1β, RSAD2, SOCS3) were upregulated among tissues/cells. Additionally, transcripts involved in metabolic processes (APOD, LRAT, RBP4) were downregulated.

**Conclusions:**

These results suggest a potential trade-off in expression of genes that regulate immunity and metabolism in birds challenged with LPS. This finding is consistent with a hypothermic response to LPS treatment in small birds. Unlike mammals, birds have nucleated RBCs, and these results support a novel transcriptomic response of avian RBCs to immune challenge.

**Electronic supplementary material:**

The online version of this article (10.1186/s12864-019-6016-3) contains supplementary material, which is available to authorized users.

## Background

Resources are rarely ubiquitous within an environment. This is especially true for organisms that migrate and/or experience seasonal alterations in life-histories. The energetically costly activities of reproduction, molt, and migration are typically cyclic [1]. In most birds, reproduction occurs on a seasonal basis when food is most abundant and generally does not conflict with other life-history events, such as molting and migration [2, 3]. Resources are also needed to combat pathogens, and immune defense is allocated strategically among life-history activities (e.g., sexual signaling, reproduction, growth; [4, 5]). These trade-offs of immune defense with other behavioral and physiological activities can serve as proximate underpinnings that shape life-history decisions [6].

The innate immune system involves rapid, indiscriminate responses [7], initiated by pattern recognition receptors that are highly conserved among vertebrates [8, 9]. The acute phase response (APR) is the first level of defense against infection as part of the innate immune system [8]. This rapid response, within hours of infection, is characterized by behavioral and physiological alterations. Stereotypical behavioral changes are known as “sickness behaviors” and include lethargy, somnolence, reduced food and water intake, and decreased activities such as grooming [10, 11]. Physiological changes occur through upregulation of genes that lead to translation of proteins that regulate fever and inflammation (interleukin-1β (IL-1β), interleukin-6 (IL-6), tumor necrosis factor-alpha (TNF-α)), and downregulation of genes associated with reproduction and growth ( [12, 13]. The APR can also be triggered by exposure to bacterial lipopolysaccharide (LPS) derived from gram-negative bacteria in a dose-dependent manner [14]. Importantly, use of LPS avoids the confounding effect of pathogen manipulation upon the immune system, such that measurement of host immune responses can be accurately quantified [10].

Most studies of the immune system have focused on adaptive immunity which takes longer to develop than innate immunity [15, 16]. Of the studies that have examined the innate immune system, and more specifically the APR, most have used mammals and domesticated birds [8, 17, 18]. Studies on free-living organisms are less common. The interdisciplinary field of ecoimmunology aims to understand how immune responses are related to host fitness, along with environmental and genetic variability, in non-model organisms [19]. However, ecoimmunology studies have typically used few markers to measure immunocompetence [20]. With newer technology, analysis of gene expression in these non-model organisms can give further understanding into the APR and trade-offs that occur with other life-history functions.

High-throughput RNA-sequencing (RNA-seq) presents advantages in non-model systems in conjunction with those model systems already in use. This method allows for the mapping and quantifying of whole transcriptomes and does not depend on an existing genomic sequence [21]. RNA-seq technology can compare expression levels in different tissues as well as facilitate identification of genes that regulate the response to infection. The relatively low cost and increased sensitivity of RNA-seq compared to other sequencing methods [21] also makes it a viable option for non-model system studies.

Previous studies using RNA-seq on zebra finch (*Taeniopygia guttata*) documented many immune-related genes, such as the major histocompatibility complex, to be constitutively expressed in a tissue-specific manner [22]. The zebra finch is one of the first birds with a fully sequenced genome [23]. One study has assessed the effect of West Nile virus (WNV) infection, in which transcriptomes were analyzed 2 days post-inoculation [24]. However, no study to date has measured rapid transcriptomic responses of birds to an immune challenge that is isolated from pathogen manipulation. The previous work in zebra finch makes it an ideal candidate organism to pinpoint functionally important immune system genes.

This study examines the transcriptomic response of hypothalamus, spleen, and red blood cells (RBCs) in zebra finch following acute challenge with LPS. We predicted an upregulation in expression of immune-regulated genes and a corresponding down-regulation of genes associated with growth and/or reproduction, which would suggest a molecular mechanism that could mediate a trade-off between immune defense and other life-history activities.

## Results

### Transcriptome and gene ontology (GO) analysis

**Fig. 1:**
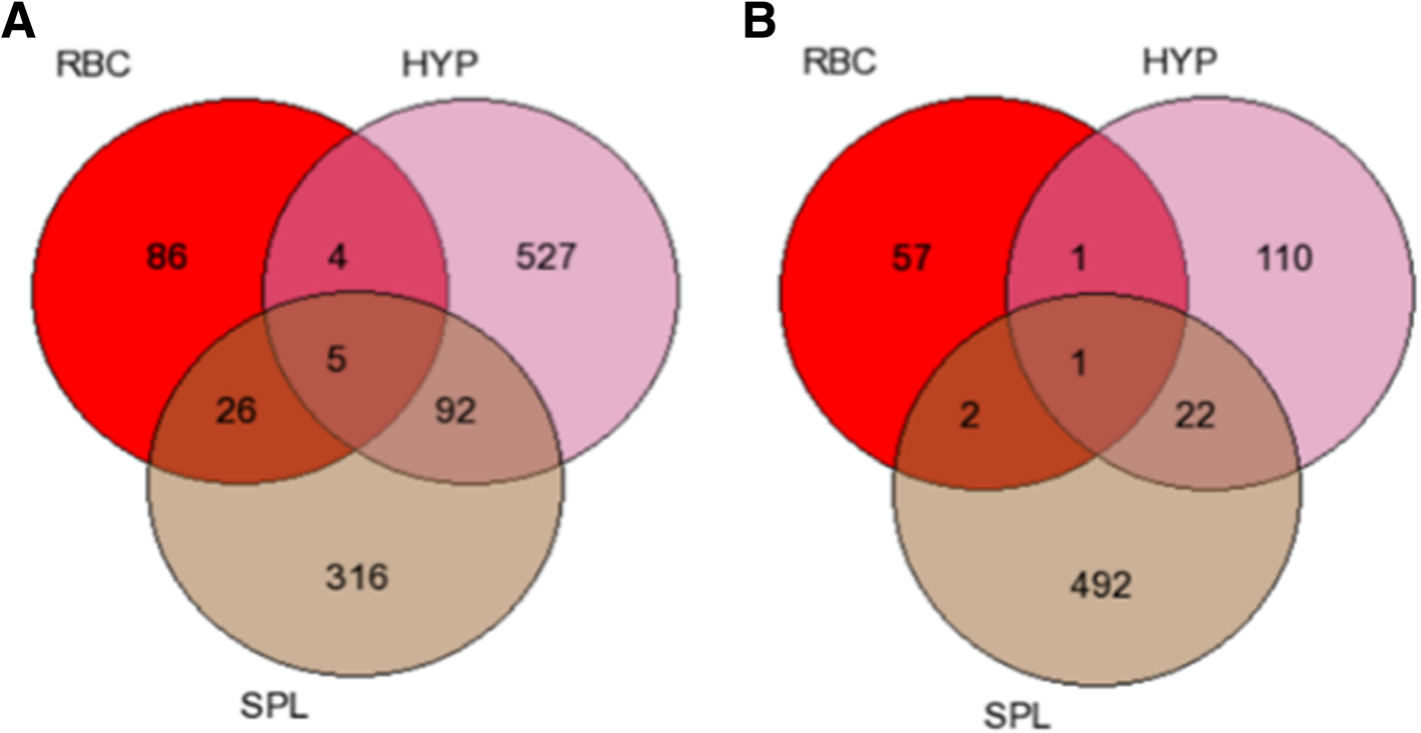
Venn diagram of number of differentially expressed genes that were annotated with gene symbols and were **a** upregulated and **b** downregulated in hypothalami (HYP), spleen (SPL), and red blood cells (RBC) after lipopolysaccharide challenge. Overlapping sets show differential expression in comparison of two or three tissues

Sequencing generated 17.5 to 63.6 million reads per sample with a mean of 32 million and standard deviation of 14.8 million. The alignment rate ranged from 69.8 to 84.4% with a mean of 80.9 across the 24 samples (Table 1). In hypothalamus, 707 genes were significantly upregulated (of which 628 had associated gene symbols) among LPS-treated birds compared with control samples. In spleen and RBCs, 564 genes (439 with associated gene symbols) and 144 genes (121 with associated gene symbols) were significantly upregulated, respectively, in LPS birds relative to controls (Additional files 1, 2 and 3; Fig. 1a, b). Additionally, 155 genes (134 with associated gene symbols) in the hypothalamus, 606 genes (517 with associated gene symbols) in the spleen, and 61 genes (all associated with gene symbols) in RBCs were shown to be significantly downregulated in LPS birds relative to control birds (Additional files 4, 5 and 6; Fig. 1b). These numbers include genes receiving overlap in differentially expressed genes (DEGs; Fig. 1a, b).

**Table 1:** Raw sequence input and alignment rate for each sample

Sample (S)	Input Reads	Aligned Reads	Alignment Rate
SAL-HYP_S1	17,513,472	14,624,051	83.50%
SAL-HYP_S2	24,094,107	19,964,614	82.90%
SAL-HYP_S3	27,047,835	22,287,716	82.40%
SAL-HYP_S4	24,981,223	21,071,895	84.40%
LPS-HYP_S5	23,162,071	19,331,652	83.50%
LPS-HYP_S6	25,034,582	20,957,262	83.70%
LPS-HYP_S7	20,983,465	17,650,778	84.10%
LPS-HYP_S8	19,537,801	16,267,282	83.30%
SAL-SPL_S9	21,331,374	17,454,947	81.80%
SAL-SPL_S10	21,437,236	17,395,505	81.10%
SAL-SPL_S11	21,433,531	17,879,727	83.40%
SAL-SPL_S12	19,190,383	15,290,676	79.70%
LPS-SPL_S13	24,568,387	20,350,638	82.80%
LPS-SPL_S14	21,547,234	17,603,507	81.70%
LPS-SPL_S15	24,649,999	20,264,221	82.20%
LPS-SPL_S16	19,107,077	13,344,122	69.80%
SAL-RBC-1_S1	57,139,999	45,112,439	79.00%
SAL-RBC-2_S2	47,612,233	37,992,062	79.80%
SAL-RBC-3_S3	51,482,969	39,720,094	77.20%
SAL-RBC-4_S4	48,209,077	37,319,383	77.40%
LPS-RBC-1_S5	51,672,988	41,299,540	79.90%
LPS-RBC-2_S6	40,124,952	32,113,483	80.00%
LPS-RBC-3_S7	63,647,656	50,109,212	78.70%
LPS-RBC-4_S8	53,561,519	42,115,678	78.60%

**Fig. 2:**
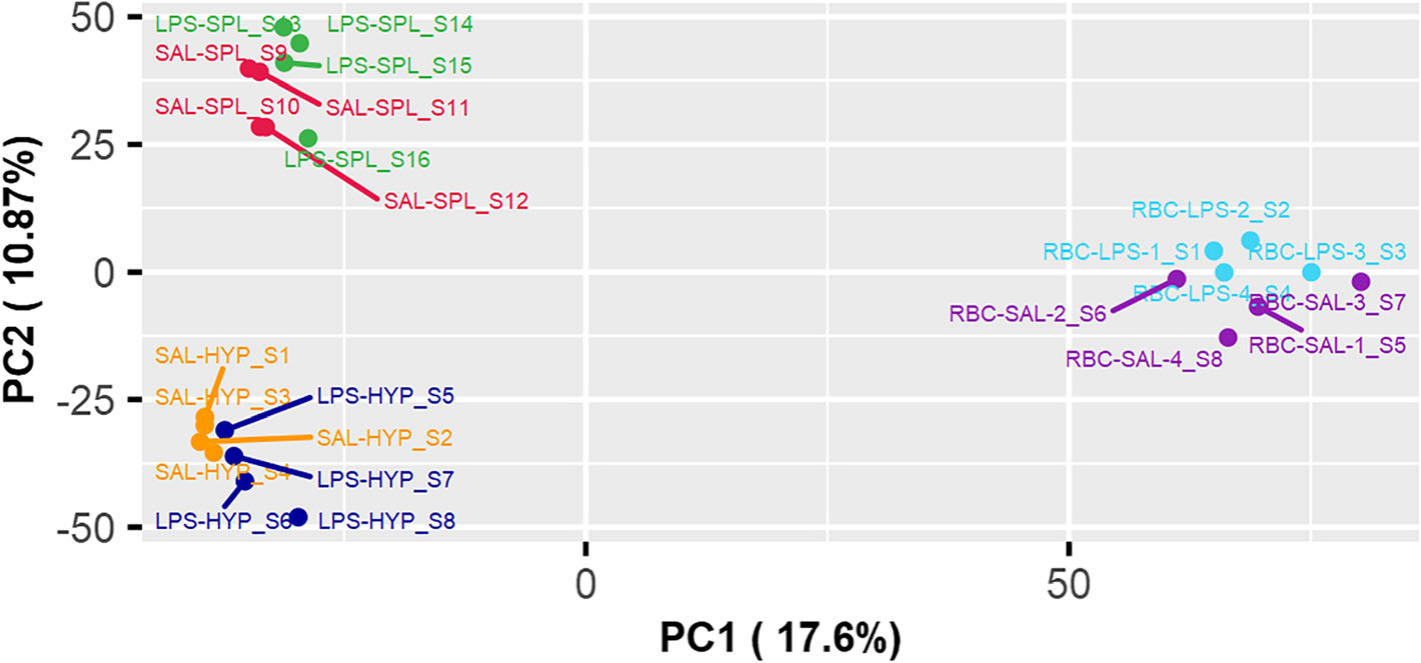
Gene expression variation of biological replicates using PCA analysis

To examine variation among replicates and tissues, principal component analysis resulted in PC1 and PC2 loadings that explained 17.6 and 10.9% of the variance in individual gene expression (measured in Fragments per Kilobase of transcript per Million mapped reads (FPKM)), respectively. Clusters separated by PC1 vs. PC2 demonstrate a separation of samples according to tissue type and treatment (Fig. 2). More specifically, PC1 delineates large variation between RBC samples and other tissues, while PC2 reflects variation among all tissue types (Fig. 2).

### Hypothalamus

GO analysis of 628 upregulated DEGs in the hypothalamus showed 92 to be involved in the stress response (Additional file 7). Several of the most upregulated genes appeared in this category were radical S-adenosyl methionine domain containing 2 (RSAD2), suppressor of cytokine signaling 3 (SOCS3), connective tissue growth factor (CTGF), G protein-coupled receptor 75 (GPR75), interferon regulatory factor 1 (IRF1), and eukaryotic translation initiation factor 4E binding protein 1 (EIF4EBP1; Additional file 7, Fig. 3). In addition, macromolecule, cellular, and protein localization and protein catabolic processes were other GO terms identified (Additional file 7, Fig. 4).

**Fig. 3:**
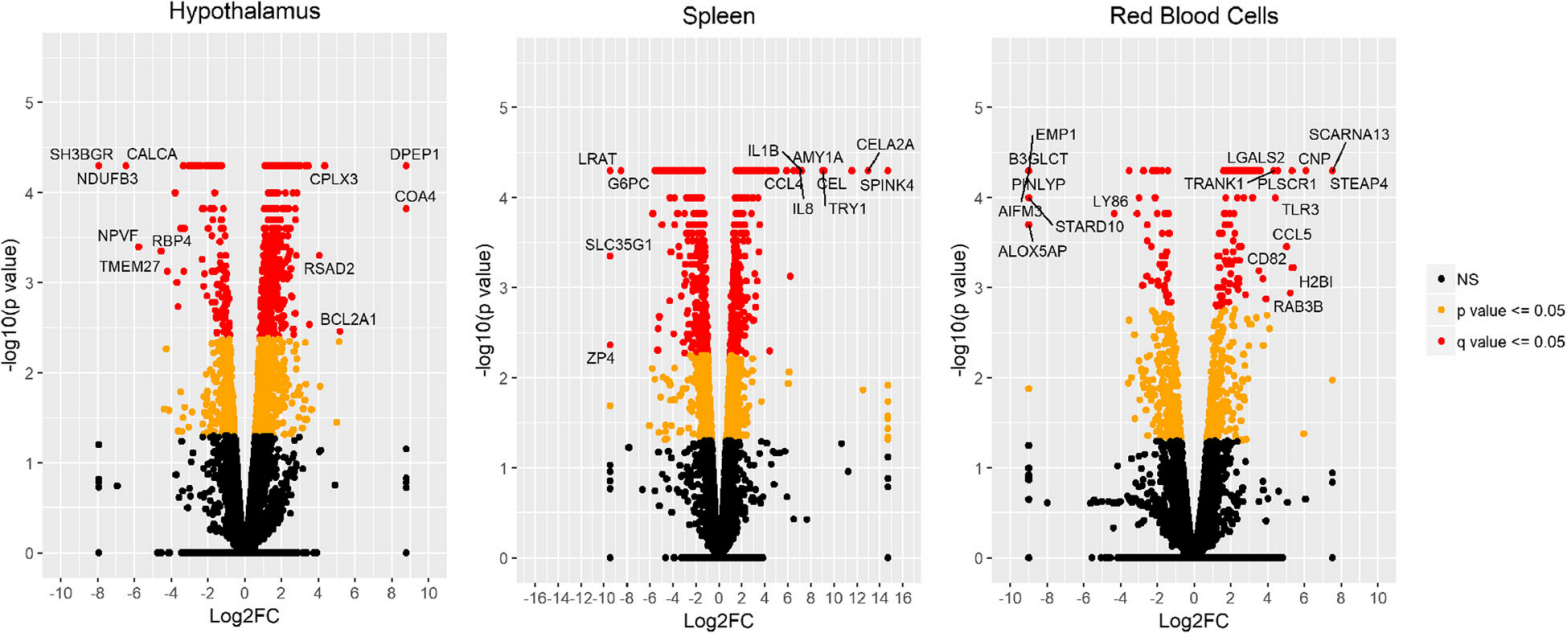
Volcano plots highlighting differentially expressed genes in orange (*p* ≤ 0.05) and red (q ≤ 0.05) for LPS vs saline control for each tissue. Top DEGs are shown

**Fig. 4:**
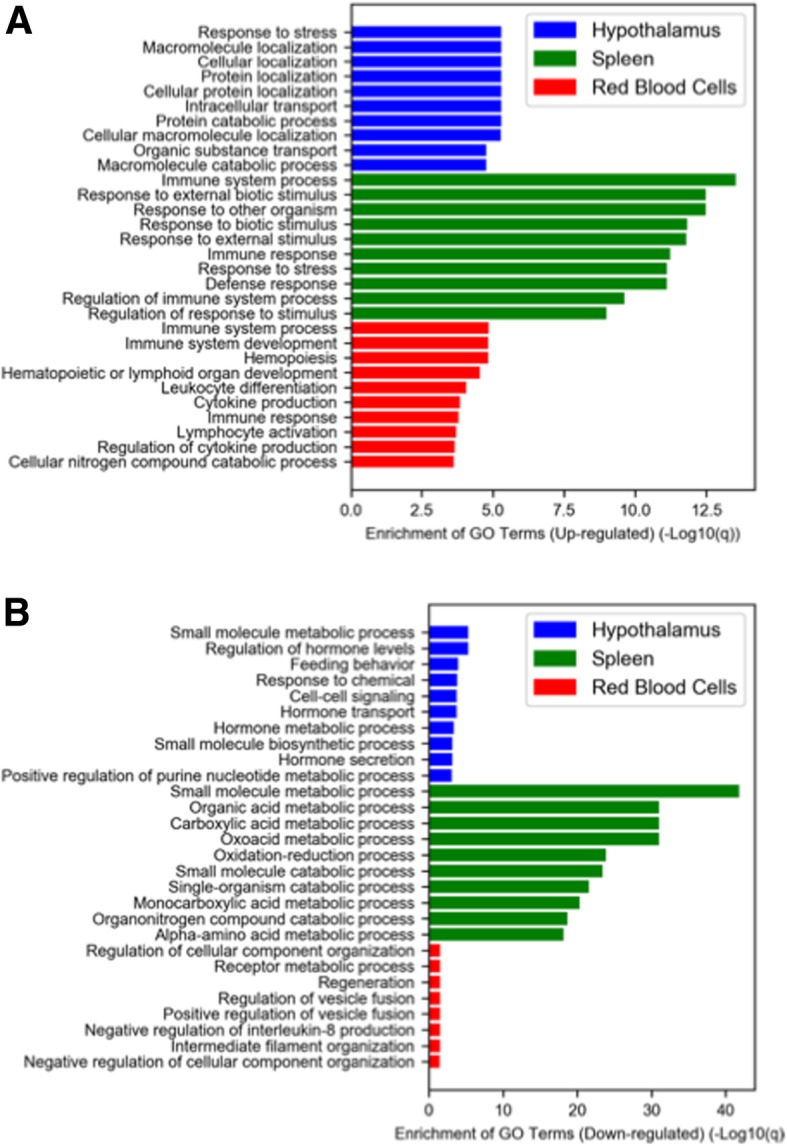
Top ten enriched Gene Ontology categories (q ≤ 0.05) for (**a**) upregulated and (**b**) downregulated genes (right) in each tissue. Note that only eight GO categories were enriched for downregulated genes of RBCs

Differential expression analysis from the transcriptomic response resulted in 134 downregulated genes that were successfully associated with gene symbols. GO analysis of the total downregulated DEGs indicated processes associated with metabolism: small molecule metabolic process, regulation of hormone levels, feeding behavior, and hormone metabolic process (Additional file 1, Fig. 4) that included calcitonin related polypeptide alpha (CALCA), retinol binding protein 4 (RBP4), carboxyl ester lipase (CEL), acetylserotonin O-methyltransferase (ASMT), phosphoenolpyruvate carboxykinase 1 (PCK1), apolipoprotein D (APOD), and tyrosine hydroxylase (TYH; Additional file 1, Fig. 3). Pro-melanin-concentrating hormone (PMCH), in addition to CALCA and TYH, were some of the genes present in the overrepresented function of feeding behavior (Additional file 7, Fig. 3). The defense gene, avian beta defensin 9 (AvBD9), was also downregulated.

### Spleen

The serine peptidase inhibitor, kazal type 4 (SPINK4) gene was the most upregulated of all genes in our global results (14.71-fold change). From GO analysis, functions associated with the 439 upregulated genes in spleen include immune system process, response to external biotic stimulus, response to other organism, response to biotic stimulus, immune response, stress response, and defense response (Additional file 7, Fig. 4). Some of the top 20 DEGs, such as ankyrin repeat and BTB domain containing 2 (ABTB2), activating transcription factor 3 (ATF3), and aldehyde dehydrogenase 1 family, member A3 (ALDH1A3), were involved in the above stress response categories. Several other DEGs were associated with more than one function, demonstrating the multi-functionality of these genes. Interleukin-1 beta (IL-1β), interleukin-8 (IL-8), C-C motif chemokine 4 homolog (CCL4) (Additional file 7, Fig. 3) are involved in the stress response, immune function, and cytokine production, while SOCS3 is involved in the stress response and cytokine production, and 2′-5′-oligoadenylate synthase-like protein 1-like (OASL) is involved in the stress response and immune function (Fig. 3).

Functions resulting from GO analysis are almost exclusively attributed to metabolic activity: small molecule, organic acid, carboxylic acid, and oxoacid metabolic processes, as well as oxidation-reduction process (Additional file 7, Fig. 4). Lecithin retinal acyltransferase (LRAT), hydroxyacid oxidase 2 (HAO2), tyrosine aminotransferase (TAT), dihydropyrimidinase (DPYS), fatty-acid amide hydrolase 1-like (FAAH), serpin family C member 1 (SERPINC1), and RBP4 are DEGs associated with metabolic process (Additional file 7, Fig. 3). Additionally, the LRAT gene experienced the largest fold-change in downregulation (− 9.51-fold change) of all DEGs across all tissues analyzed.

### Red blood cells

From the GO analysis on the 121 upregulated DEGs of the RBCs, immune and cytokine function (e.g. innate immune response, leukocyte activation, cytokine production, type I interferon production), immune system development, hematopoesis, hematopoetic or lymphoid organ development, as well as stress response, was observed (Additional file 7, Fig. 4). C-C motif chemokine ligand 5 (CCL5), toll-like receptor 3 (TLR3), leucine-rich repeat kinase 1 (LRRK1), serum amyloid A like 1 (SAAL1), mitogen-activated protein kinase kinase kinase 8 (MAP3K8), and interferon induced with helicase C domain 1 (IFIH1) were some of the genes involved with immune and cytokine functions (Additional file 7, Fig. 3).

There were 61 significantly downregulated genes in the RBCs. GO analysis indicated the following functional terms: regulation of cellular component organization, receptor metabolic process, regeneration, regulation of vesicle function, and negative regulation of interleukin 8 (IL-8; Additional file 7, Fig. 4). Annexin A1 isoform p37-like (ANXA1) and arrestin beta 1 (ARRB1) were among the most downregulated genes that contribute to the negative regulation of IL-8 (Additional file 7, Fig. 3).

### Shared genes

Five genes were significantly upregulated in all three tissues tested (Table 2). Two of the five common genes— CCL5 and RSAD2—were reported among the top 30 overrepresented functional categories. Additionally, a single gene, glutathione S-transferase class-alpha variant 2 (GSTA2), was downregulated in all three tissues. The GSTA2 gene is involved in the metabolic process according to its GO annotation (Table 2).

**Table 2:** List of upregulated and downregulated transcripts found in all three tissues, following lipopolysaccharide challenge. Column headers show the Ensembl transcript ID tag, gene symbol, full gene name as description, the log base 2 transformed fold change (FC) for hypothalami (HYP), spleen (SPL), and red blood cells (RBC) compared to controls, respectively, and the corresponding *q*-value of the gene for each tissue

Ensembl Transcript ID	Gene Symbol	HYP log_2_(FC)	*q*-value	SPL log_2_(FC)	*q*-value	RBC log_2_(FC)	*q-*value
Upregulated							
ENSTGUG00000003209	CCL5	2.41	0.004	1.38	0.031	5.04	0.017
ENSTGUG00000012992	RSAD2	4.05	0.011	3.86	0.001	2.89	0.004
ENSTGUG00000001423	STEAP4	1.53	0.045	4.52	0.001	7.53	0.004
ENSTGUG00000004451	TOR1B	1.39	0.010	2.19	0.001	2.77	0.004
ENSTGUG00000005628	ZNFX1	1.87	0.006	2.77	0.001	1.79	0.023
Downregulated							
ENSTGUG00000013481	GSTA2	−1.23	0.003	−1.31	0.049	−3.51	0.038

## Discussion

The aims of this study were to determine the transcriptomic response of zebra finch to LPS challenge using RNA-seq and to assess whether trade-offs between immune function and other life-history activities (growth/reproduction) could be recapitulated at the molecular level. However, gene transcripts that directly influenced growth or reproduction were not transcriptionally down-regulated in response to LPS. Rather, transcripts responsible for regulating metabolism exhibited down-regulation. Because we examined the genomic response to LPS on an acute time scale (2 h post-challenge), it is possible that genes responsible for controlling growth and/or reproduction may occur at a later time frame following immune challenge. For example, in white-crowned sparrows (*Zonotrichia leucophyrs)*, the hypothalamic-pituitary-gonadal (HPG) axis is suppressed 24 h after LPS injection as measured by a reduction in plasma luteinizing hormone concentration [25].

Genes involved with the stress response were upregulated within the hypothalamus in response to LPS challenge. This response is consistent with rapid activation of the hypothalamic-pituitary-adrenal (HPA) axis that occurs during the APR, including in birds [25, 26]. Given the well-known function of the spleen in regulating immunity, it was not surprising that immune function and cytokine production genes were activated in the spleen. However, the increased expression of immune-related and cytokine production-related genes in avian RBCs indicates a novel function of nucleated RBCs. Most studies to date have assumed that RBCs play little or no role in regulating immunity, but only a few have examined non-mammalian RBCs [27, 28].

The downregulated transcripts of the hypothalamus and spleen imply a reduction in metabolism based on the Gene Ontology (GO) analysis (Additional file 7; Fig. 4). Although many endotherms exhibit fever when challenged with LPS, in small songbirds, such as the zebra finch, hypothermia results with a concurrent decrease in metabolic rate [14], which likely explains why genes associated with metabolism were down-regulated rather than up-regulated. It is hypothesized that in small endotherms, the heat loss from a fever response would be more detrimental than the purported advantages [29]. Hyperthermia, experienced by most of the larger endotherms (pigs, goats, and chickens), and hypothermia in small birds, is thought to be regulated by the same inflammatory and stress signals that trigger fever [30]. In the hypothalamus, feeding behavior and hormone-related processes were also down-regulated. A decrease in feeding behavior (anorexia) is a common occurrence observed in animals injected with LPS [8, 25].

Of the total number of transcripts differentially expressed in each tissue, only six were shared among all three, and included five upregulated genes and one downregulated gene (Fig. 1a, b; Table 2). The majority of differentially expressed genes (DEGs) were tissue specific, with the hypothalamus experiencing the largest shift in unique upregulated DEGs and spleen having the most downregulated. These data suggest that different tissues have a specialized role when responding to an immune challenge, although the processes they are involved with regulating could be similar. The most DEGs within overrepresented GO functional categories are the most informative for the study and were chosen for further examination within those functions.

### Hypothalamus

The hypothalamus plays an integral role in homeostasis by regulating body temperature, appetite, reproductive behaviors, and circadian rhythms. The APR, triggered by LPS treatment, activates the HPA axis and suppresses the HPG axis via stimulation by inflammatory cytokines such as IL-1β [8, 31].

Some of the notable genes involved with stress response were RSAD2, SOCS3, and EIF4EBP1. Also known as viperin, RSAD2 is upregulated in response to H5N1 avian flu [32] and dengue infections [33] through involvement in interferon and cytokine signaling pathways. RSAD2 is upregulated in chicken kidney samples following avian infectious bronchitis virus exposure [34], suggesting that RSAD2 might be induced during early infection. SOCS3 and EIF4EBP1 are associated with the inhibition of interferon production via a negative feedback loop that prevents interferon and other inflammatory cytokines from inducing immunopathology [35]. SOCS3 was upregulated by LPS challenge with a 3.462-fold change increase compared to control samples. Ranaware et al. [32] reported a 4.2-fold change increase following H5N1infection in chicken lung tissue. It is also possible that a virus, such as H5N1 or influenza A, may evade the host’s immune response by inducing cytokine suppressors (SOCS1 and SOCS3), that in turn inhibit interferon and toll-like receptor signaling [36, 37]. EIF4EBP1 augments the innate immune system as a translational repressor of interferon production [38]. EIF4EBP1 acts by repressing expression of interferon regulatory factor 7 (IRF7), thereby repressing interferon production [39]. Without EIF4EBP1 repression, IRF7 triggers interferon production, creating a positive feedback loop for further IRF7 and interferon production. IRF7 was not significantly upregulated in these samples (0.964-fold change, *q*-value 0.191), and interferon was not present. However, there was upregulation in IRF1 (2.975-fold change; Additional file 2). The products of IRF1 also trigger an interferon response during early infection. A moderate upregulation in IRF1 transcription was seen in chicken lung following H9N2 infection [32] and IRF1 knock-out mice cannot survive West Nile virus infection [40]. In hypothalamus, IL-1β was not significantly upregulated (*q*-value of 0.053), but this does not discount a possible biological significance, especially when examining the fold change (5.132) and results found in the other tissues examined (fold change value 7.184 in spleen and 2.129 in RBC).

Genes associated with metabolism and cellular and protein localization were down-regulated in LPS-treated birds. The CALCA gene encodes several peptide hormones, including calcitonin and calcitonin-gene-related peptide (CGRP), that are linked to glucose and lipid metabolism [41] . CGRP is also involved in controlling inflammation through anti-inflammatory actions [42, 43]. In chronic inflammation disorders, such as inflammatory response syndrome in dogs, CALCA is upregulated [44], where it inhibits tumor necrosis factor (TNF) and interleukin 12 (IL-12) cytokines [42]. Tsujikawa et al. [45] documented this anti-inflammatory effect 24 h after LPS treatment in mice, where cytokine levels were highest 3–6 h post-treatment.

Another downregulated gene that influences both metabolism and inflammation is RBP4, a negative acute phase inflammatory reactant. Decreased transcription in favor of the amino acids being used for producing positive acute phase reactants, such as IL-1β and TNF, that also inhibit RBP4 likely potentiates the APR [46–48]. Metabolic GO terms associated with this gene include ‘gluconeogenesis’ and ‘positive regulation of insulin secretion,’ and it has been linked with human obesity [49]. The RBP4 protein is required for the transport and utilization of retinol (vitamin A) from the liver to peripheral tissues [50] where retinol derivatives are involved in lipid metabolism [51]. However, retinol has been shown to enhance recovery from infection via actions in lymphoid tissues and its role in immune cell development (neutrophils, macrophages, natural killer cells; [52]). During the APR, already circulating retinol can be utilized, but less will be transported from the liver due to the downregulation of RBP4 transcription [50]. Consequently, the immune system of malnourished individuals, with less circulating retinol before infection, may have a decreased ability to combat infection.

There were also downregulated genes involved with mediating feeding behavior. PMCH encodes melanin-concentrating hormone (MCH), with confirmed activity in appetite control by the use of MCH agonists and antagonists [53]. Saito et al. [54] and Adams et al. [55] demonstrated the role of this hormone in olfaction, where the downregulation or deletion of PMCH impaired food-seeking behaviors. CALCA and TYH are also in this category for their metabolic cascades driving energy homeostasis by CALCA [41] and brain function products by TYH [56].

The differing roles of genes that were up- or downregulated in the hypothalamus following LPS treatment reflects a potential trade-off between functions. Several of the most upregulated genes code for proteins that influence cytokine pathways. Control of cytokine activity is evident in our results through the upregulation of the cytokine inhibitors SOCS3 and IEF4EBP1. All of the downregulated genes discussed here participate in metabolism and/or feeding behaviors. CALCA has anti-inflammatory actions [42, 43] which are depressed during the APR in this study. The reduction in feeding behaviors by the downregulation of CALCA, PMCH, and TYH may lower the risk of being a predatory target when searching for food when the animal is not in optimal health. The TYH enzyme product facilitates catecholamine synthesis (dopamine, epinephrine, norepinephrine), further affecting brain functions such as attention, memory, and cognition [56]. The above results suggest a physiological shift towards combating illness, and a reduction in unnecessary energy expenditure and behaviors that could increase risk of predation.

### Spleen

One of the largest organs of the lymphatic system, the spleen filters fluids in the body, including blood. During filtration, excess blood can be stored, old RBCs removed, and iron recycled [57]. The spleen also plays a prominent role in the peripheral immune system. When macrophages in the spleen interact with bacterial or viral components, the innate immune system is triggered. Additionally, the spleen is involved in the adaptive immune system through B cell production of antibodies and T cell activation [58].

In response to immune challenge by LPS, the spleen experienced an upregulation in genes involved in immune function, cytokine production, and stress response (Additional file 7, Fig. 4). This finding is consistent with the role that the spleen plays in responding to bacterial infections. The potent cytokine gene, IL-1β, is involved in the stress response, immune function, and cytokine production GO function categories. IL-1β is one of the most studied pro-inflammatory cytokines for its crucial role in initiating and regulating the immune system, and has been well documented in chickens by LPS injection [59], infectious bursal disease [60], and different bacterial infections [61, 62]. In wild birds that act as natural reservoirs for disease and encounter domesticated animals, IL-1β is less understood. Park et al. [63] showed that house finch splenocytes had several cytokines significantly induced by IL-1β, including interleukin 2 (IL-2), interleukin 10 (IL-10), and chemokine C-X-C motif ligand 1 (CXCL1).

Two chemokines, IL-8 and CCL4, were also upregulated. IL-8 products induce lysosomal enzyme release from neutrophils and chemotactic activity for basophils and adaptive T cells [64]. IL-8 gene is greatly stimulated by IL-1β, but not by interferon [65] and is elevated in respiratory distress syndrome in humans [66]. CCL4 also encodes products with chemotactic activity. CCL4 attracts natural killer cells, monocytes, and in later adaptive responses, T cells [67]. Of interest is the change in expression levels of CCL4 with H5N1 infection. Compared to seasonal influenza viruses, such as H1N1 and H3N2, CCL4 is more strongly induced by H5N1 [68]. Cheung et al. [69] even reported the downregulation of CCL4 in human samples with H1N1 infection. This relationship of H5N1 and CCL4 may have additional implications for wild and domestic avian species.

A gene involved with the stress response in spleen in response to LPS was ALDH1A3. This gene produces the rate limiting dehydrogenase enzyme for converting retinol (vitamin A) to retinoic acid, a hormone-like metabolite with the ability to modulate immune responses by promoting inflammation [70]. Retinoic acid activates and increases cytokine production in the innate immune system and is used by dendritic, B, and T cells in the adaptive immune system [71]. Increased conversion of retinol by the upregulation of ALDH1A3 also decreases the limited serum retinol resulting from the downregulation in transcription of its transporter, RBP4. Without this transporter, retinol remains stored in the liver and unusable in other parts of the body [52]. The limit on retinol is further compounded by the fact RBP4 is downregulated in both the hypothalamus and spleen.

SOCS3, involved in both the stress response and cytokine production, was upregulated in the hypothalamus and spleen (Additional file 7, Fig. 3). SOCS3 helps regulate the immune system response by preventing the induction of immunopathology from cytokines [35]. Cytokine genes were among the most upregulated in spleen compared to the hypothalamus, suggesting that increased expression of SOCS3 facilitates the dampening of splenic inflammatory responses from cytokines.

LPS induced a down-regulation of splenic genes that regulate metabolism. One of these, LRAT, is involved in retinol metabolism as discussed above. Specifically, it encodes the predominant enzyme for esterification of retinol [72, 73]. LRAT negatively regulates retinoic acid biosynthesis [74]. The downregulation of LRAT, along with the upregulation of ALDH1A3 (see above), can result in increased biosynthesis of retinoic acid. The increased presence of retinoic acid further suggests the importance of retinoic acid in inflammation. As in the hypothalamus, RBP4 was downregulated in the spleen.

Among other downregulated genes involved in metabolism, HAO2 encodes an enzyme that mediates the oxidation of fatty acids [75, 76]. Likewise, the hydrolase encoding gene, FAAH, is involved in the hydrolysis of 2-arachidonoylglycerol, a lipid that stimulates phagocytic activities in macrophages during a defense response [77]. Also, by the downregulation of TAT, the amino acid tyrosine is not catabolized [78, 79], possibly making the amino acid further available for cytokine production. Another downregulated gene, DPYS, encodes a rate-limiting enzyme that plays a role in pyrimidine catabolism [80]. Inhibiting these genes reduces degradation of materials needed by the immune response, such as amino acids and immune system mediators.

In spleen, there was polarized expression of immune system genes and metabolic genes as in hypothalamus. The spleen shared the upregulation in the cytokine suppressor, SOCS3, with the hypothalamus. RBP4, the negative acute phase reactant, was a downregulated gene shared with the hypothalamus, in the metabolic functional category. From specific functions of genes in the metabolic category, there was a reduction in catabolic processes of fatty acids, amino acids, and pyrimidines. The most differentially expressed gene in either direction was also found within the spleen: SPINK4 (14.710-fold change) and LRAT (− 9.510-fold change). The downregulation of LRAT, which normally inhibits retinoic acid biosynthesis, along with the upregulation of ALDH1A3, further suggests the importance of retinoic acid to the immune system. The known functions of the spleen, compared to the hypothalamus, and the absence of behavioral-based genes is expected (Fig. 4).

### Red blood cells

In contrast to mammalian RBCs, avian, reptilian, and amphibian RBCs contain a nucleus [81]. The best reported defense that anucleated RBCs have against pathogens is the release of reactive oxygen species from hemoglobin when the cell is lysed, breaking down lipids, proteins, and DNA of nearby pathogens [82]. Little attention has been given to the function of nucleated RBCs. It has been assumed these nucleated RBCs merely participate in gas exchange, with little or no protein synthesis, like mammalian anucleated RBCs [83]. Studies using avian whole blood show an immune response [84, 85] but the results cannot differentiate between RBC and leukocyte activity.

More recently, a few studies examined the activity of nucleated RBCs in immune function. There is now evidence of active transcriptional machinery within these cells that do react to various stimuli. A differential response of genes involved in the immune system and metabolic processes was shown in trout and chicken cultured RBCs [28]. To our knowledge this is the first study to use RNA-seq for quantitative analysis of nucleated RBCs to immune challenge in a wild avian species.

The reaction of the nucleated RBCs in the zebra finch to LPS involve genes associated with the immune response. Genes that regulate inflammation, cytokine production, and the stress response were upregulated within top DEGs (Additional file 7, Fig. 4). One of these, the chemokine CCL5, is expressed early in an immune response, activated through signaling by the presence of pro-inflammatory cytokines and by contact with pathogens [86, 87]. Elevated expression of CCL5 has been noted in hantavirus, reovirus, adenovirus, and influenza virus (H5N1) infections, but severe acute respiratory syndrome (SARS) coronavirus is an especially strong inducer in human lung tissue [87].

Three upregulated genes are involved in a cascade of signaling: MAP3K8, TLR3, and IFIH1. MAP3K8, activated by IL-1β, is critically involved in TLR signaling [88]. TLR3 propagates signals after being induced by interferons and cytokines and is involved in RIG-I and MDA5 pathways [89]. Upregulation of TLR and IFIH1 (MDA5) is documented in chickens infected with H5N1, low pathogenic avian influenza (LPAIV) H7N1, and avian Tembusu virus (ATMUV) that cause great economic loss and present a danger to humans [90]. Once activated, the gene encoding MDA5 initiates further signal transduction for cytokine secretion. MDA5 is the primary influenza A virus sensor for chickens [89]. The upregulation of TLR3 is one gene that can be used to assess RIG-I or MDA5 expression, depending on the avian species.

Few functional categories were produced by GO analysis of the downregulated RBC genes, and few genes represented these functions. Out of 61 downregulated genes, the functional category of regulation of cellular component organization only contained 12 genes from the DEG list. Given that many of the genes in zebra finch are not annotated thoroughly, this could lend to a lack in functional categorization.

Two genes were involved in the negative regulation of IL-8. IL-8 has chemotactic activities for basophils, as well as stimulating phospholipase enzymes that interact with neutrophils [64, 91]. Both downregulated genes in this category have anti-inflammatory activity. ANXA1 inhibits phospholipase, interfering with IL-8 downstream mediation of inflammation. ARRB1 inhibits upstream processes in TLR signaling that lead to IL-8 expression [92, 93].

These novel findings show that there are transcriptomic adjustments during the APR of the immune response. In fact, the six most upregulated DEGs are involved in the immune system and/or cytokine production. The upregulation of CCL5 in this these cells may be advantageous because of the gene product’s role in leukocyte recruitment. Despite there being few downregulated genes represented in the GO functional categories, ANXA1 and ARRB1 are involved in the negative regulation of IL-8. With dampened expression of ANXA1 and ARRB1, IL-8 involved signaling is not inhibited. More can be elucidated about the complete transcriptome changes from immune challenge by examining the other 115 upregulated and 58 downregulated genes.

### Shared genes

Of the five genes shared between all three tissues, only two contributed to the functions produced by GO analysis: CCL5 and RSAD. While not within the top most upregulated in all tissues, they were both statistically significantly expressed. The first of these genes, CCL5, as discussed, is a chemokine that regulates leukocyte trafficking [87]. It has also been highly conserved during evolution, with sequence homology in mammals, fish, and birds [86]. The second, RSAD2, regulates interferon and cytokine signaling pathways. The exclusion of the other genes was based on their associated GO terms.

The single downregulated gene that was shared, GSTA2, has the biological process GO term: ‘metabolic process.’ The encoded enzyme is reported to be involved in cellular detoxification and excretion of several xenobiotic substances, with strong implications for ingestion of mycotoxin aflatoxin B1 in turkeys [94]. Turkeys are especially susceptible to the toxin because GSTA2 and the enzyme product are unresponsive to aflatoxin B1. IL-6 was significantly upregulated in the spleen compared to controls (1.834-fold change, *q*-value 0.030). This is further explained by GSTA2’s anti-inflammatory properties and activation by glucocorticoids. Interestingly, GSTA2 is associated with plumage dichromatism; GSTA2 and APOD are involved with carotenoid uptake, binding, and deposition in pheasants [95]. In this study, APOD was downregulated in the hypothalamus (− 3.050-fold change, *q*-value 0.002). The role of GSTA2 in carotenoid processes suggests a downregulation much like with RBP4 and LRAT, by reducing interference in the use of retinoic acid.

### Ecological impact

Given these results we have a better understanding of the global transcriptomic changes in the hypothalamus, spleen, and nucleated RBCs of zebra finch following activation of the APR by bacterial LPS. This study provides insight into physiological changes during the APR, with many genes involved in the immune system being upregulated and genes in metabolic pathways being downregulated. Because the environment of free-living organisms is widely variable in resources and conditions, it is important to understand these potential trade-offs and implications on seasonally important functions, such as migration, reproduction, and growth. For example, parental care has been shown to decrease in house sparrows (*Passer domesticus*) during illness [96], as well as LPS injection reducing territorial aggression in white-crowned sparrows [25].

### Future work

#### White blood cells

Comparing transcriptome changes of RBCs and WBCs will give further insight into the APR of zebra finch. However, complications with obtaining an appropriate amount of RNA for sequencing prevented that analysis in this study. Although RNA-seq requires much less RNA than other methods [21], the concentration of WBCs in whole blood is low. Combined with the size of the zebra finch, other methods or techniques will be needed to accomplish this in the future.

#### RT-PCR validation

Further validation of these results can be done through RT-PCR. This would be best accomplished with multiple gene primers based on the sheer number of DEGs in our data. Custom primer design is recommended.

## Conclusions

This study provides insight into the rapid molecular responses that occur during immune system activation in a passerine bird species. The APR is an evolutionarily conserved rapid response to eliminate and control infection, and within 2 hours of LPS administration there is upregulation of immune genes and a downregulation in genes putatively involved with metabolic pathways in the hypothalamus and spleen. The upregulation in immune related genes in the hypothalamus, spleen, and red blood cells supports part of the hypothesized transcriptomic response to immune challenge in zebra finch. However, the current results can only *suggest* a trade-off in immune function and other important aspects of life. Further research is needed to examine this potential trade-off using experimental methods.

By using LPS, a component of gram-negative bacteria, pathogen manipulation is avoided. Tissue specificity is also shown by the DEGs not shared between and among tissues (Figs. 1 and 2). Of the total DEGs, only five upregulated and one downregulated gene were shared. We additionally present novel insight into transcriptional changes in nucleated RBCs of a non-model avian organism. These findings on the immunological response of avian RBCs will lay a framework for further investigations into the function of nucleated RBCs, a topic that to date has been poorly studied.

## Methods

### Animals

Male zebra finch (*Taeniopygia guttata*) taken from our breeding colony were housed in individual cages (16.5 × 11.8 × 22, Petsmart Co.) in the animal facility at Western Kentucky University for 7 days. Food (Finch and Canary Breeding and Molting Seed Blend, Lady Gouldian Finch, Irvine, CA; lettuce/cabbage leaves) and water were provided ad libitum. Animals were exposed to a 12 h light: 12 h dark photoperiod (lights on at 0700) and an ambient temperature of 23 ± 1 °C. Between 4 and 5 h after lights on, birds were quickly captured (under 30 s) and given an i.p. injection of either bacterial lipopolysaccharide (LPS; 2 mg/kg of BW; *n* = 8 *Escherichia coli*; serotype 026:B6; Sigma-L8274) dissolved in 0.9% saline (vehicle) or vehicle alone (*n* = 8). Two hours later, animals were euthanized using isoflurane until unconscious (< 20 s) and then rapidly decapitated. Brain and spleen were collected from half of the treatment group (*n* = 4) and half of the control group (*n* = 4) and immediately placed in RNA*later* (AM7021; Thermo Fischer Scientific). Tissues were stored in RNA*later* at − 20 °C until processed. The hypothalamus was dissected out of the brain before RNA isolation (see below). Whole trunk blood was collected from the remaining four treatment and four control birds and placed into tubes containing EDTA and kept on ice until processed (see “Blood” below).

### Blood

EDTA-treated blood was layered on top of a single step density gradient medium (PolymorphPrep; Axis-Shield) consisting of 1-part blood: 2-parts gradient medium and then further processed following the manufacturer’s protocol. Layers of peripheral blood mononuclear cells (PBMC), polymorphonuclear leukocytes (PMN), and white blood cells (WBCs) were extracted and pooled per individual animal. Slides were prepared from erythrocyte (red blood cells; RBCs) and PBMC/PMN samples and stained using Hemacolor Rapid (Millipore Sigma) following the manufacturer’s protocol. Each slide was surveyed under 400 x magnification for 10 min to assess for contamination before RNA isolation.

### RNA isolation

RNA was isolated from PBMC/PMNs and RBCs using Trizol (Ambion) lysing and a RNeasy Mini Kit (Qiagen). The hypothalamus and spleen were homogenized and RNA was extracted using a RNeasy Mini Kit (Qiagen) following the manufacturer’s instructions. RNA yield and purity were assessed using a NanoDrop N-D 2000 spectrophotometer (Thermo Fischer Scientific) and an Agilent 2100 Bioanalyzer (Agilent), respectively. Total RNA concentrations ranged from 10.0 to 53.6 ng/μl for hypothalami, 17.2 to 77.2 ng/μl for spleen, and 4.6 to 20.8 ng/μl for RBCs. RNA Integrity Numbers (RIN) were > 7.5. Unfortunately, PBMC/PMNs yielded RNA concentrations that were too low for sequencing purposes. Low RNA yield can be attributed to the low concentration of WBCs in whole blood and the small volume of whole blood we were able to obtain from zebra finch (typically < 150 μl).

### Library preparation and sequencing

Samples were analyzed at the University of Louisville Genomics Core (Louisville, KY) for library preparation and sequencing. SMART-Seq v4 Ultra Low Input RNA kits (Takara Bio USA, Inc.) were used to prepare cDNA libraries. Samples were barcoded with Illumina TruSeq Adapters. After library clean-up using Agencourt AMPure XP Beads, quality was assessed on an Agilent Bioanalyzer using the Agilent DNA 1000 Kit. This confirmed the final fragment size for all samples to be approximately 400 bp, as expected from the protocol. Samples were sequenced twice on an Illumina NextSeq 500 sequencer, with four biological replicates and four lanes per replicate.

### Bioinformatics analysis

Bioinformatics analysis was performed at the Kentucky Biomedical Research Infrastructure Network (KBRIN) Bioinformatics Core at the University of Louisville Genomics Core. Raw sequencing files were downloaded from Illumina’s BaseSpace. Quality scores for raw sequences were sufficiently high, above the recommended score of 20 (base call accuracy of 99%), to proceed with analysis [97, 98]. The Tuxedo Suite pipeline was used for analysis of data [99]. High quality single-end reads (152 million for hypothalamus, 140 million for spleen, and 326 million for RBCs) were successfully mapped to the zebra finch reference genome (taeGut3.2.4.84) from Ensembl (http://www.ensembl.org; [100]) using the TopHat2 (v. 2.0.13) tool. Differential expression between LPS and saline conditions was determined from fragments per kilobase million (FPKM) normalized read counts using Cuffdiff2 (v. 2.2.1) of the Tuxedo Suite programs and log base 2 transformed. The replicates were handled as a group (LPS-injected birds were treated as one group and saline-injected birds in another group). The Cuffdiff2 program calculates the mean value and variance as it assigns *p*-values and adjusted *p*-values. All fold change expression values presented from this study are provided as log base 2. Genes with Benjamini-Hochberg false discovery rate (FDR) (*q*-value) ≤ 0.05 and a log base 2 transformed fold change ≥1 were considered significantly differentially expressed.

To assess variation within groups and among tissues, we conducted a principal component analysis using normalized read counts (FPKM) for 18,168 tested genes locations as input using the R statistical function ‘prcomp.’

### Gene ontology analysis

Gene annotations came from Ensembl BioMarts as part of the KBRIN Bioinformatics Core analysis [101–103]. Transcripts with differential expression from sequence code but no known gene ID were excluded from further analysis. Gene ontology (GO) enrichment analysis was performed on differentially expressed genes (DEGs) using the ShinyGO web-based tool (http://bioinformatics.sdstate.edu/go/) (v. 0.6). GO functional categories falling under biological processes, with a *q*-value ≤0.05 following hypergeometric testing, were considered significantly overrepresented. In the hierarchy of GO, a gene can be represented in more than one category because of the functional versatility of genes, but only once within each category. Likewise, genes appear in the categories represented by both the specific (child term) and broad (parent term) categories under these biological processes.

## Additional files

Additional file 1: Upregulated DEGs in hypothalamus. Raw DEG data for upregulated genes in hypothalamus. (XLSX 344 kb)

Additional file 2: Upregulated DEGs in spleen. Raw DEG data for upregulated genes in spleen. (XLSX 267 kb)

Additional file 3: Upregulated DEGs in red blood cells. Raw DEG data for upregulated genes in red blood cells. (XLSX 42 kb)

Additional file 4: Downregulated DEGs in hypothalamus. Raw DEG data for downregulated genes in hypothalamus. (XLSX 141 kb)

Additional file 5: Downregulated DEGs in spleen. Raw DEG data for downregulated genes in spleen. (XLSX 253 kb)

Additional file 6: Downregulated DEGs in red blood cells. Raw DEG data for downregulated genes in red blood cells. (XLSX 26 kb)

Additional file 7: Gene ontology analyses for each tissue. Lists of 30 most significantly overrepresented GO terms for upregulated and downregulated genes of the hypothalamus, spleen, and RBCs. (DOCX 32 kb)

## Data Availability

Raw sequencing data and analyses for this study are available at GEO (GSE121348): https://www.ncbi.nlm.nih.gov/geo/query/acc.cgi?acc=GSE121348.

## Abbreviations

ABTB2: Ankyrin repeat and BTB domain containing 2
ALDH1A3: Aldehyde dehydrogenase 1 family, member A
ANXA1: Annexin A1 isoform p37-like
APOD: Apolipoprotein D
APR: Acute phase response
ARRB1: Arrestin beta 1
ASMT: Acetylserotonin O-methyltransferase
ATF3: Activating transcription factor 3
ATMUV: Avian Tembusu virus
AvBD9: Avian beta defensin 9
BW: Body weight
CALCA: Calcitonin related polypeptide alpha
CCL4: C-C motif chemokine 4 homolog
CEL: Carboxyl ester lipase
CTGF: Connective tissue growth factor
DEGs: Differentially expressed genes
DHX58: DEXH (Asp-Glu-X-His) box polypeptide 58
DNA: Deoxyribonucleic acid
DPYS: Dihydropyrimidinase
EDTA: Ethylenediaminetetraacetic acid
EIF4EBP1: Eukaryotic translation initiation factor 4E binding protein 1
FAAH: Fatty-acid amide hydrolase 1-like
FDR: False discovery rate
FPKM: Fragments per Kilobase of transcript per Million mapped reads
GO: Gene ontology
GPR75: G protein-coupled receptor 75
GSTA2: Glutathione S-transferase class-alpha variant 2
H1N1: Influenza A virus subtype H1N1
H3N2: Influenza A virus subtype H3N2
H5N1: Influenza A virus subtype H5N1
H7N1: Influenza A virus subtype H7N1
HAO2: Hydroxyacid oxidase 2
HPA: Hypothalamic-pituitary-adrenal
HPG: Hypothalamic-pituitary-gonadal
IFIH1: Interferon induced with helicase C domain 1
IL-1β: Interleukin-1-beta
IL-6: Interleukin-6
IL-8: Interleukin-8
IRF1: Interferon regulatory factor 1
JUN: Transcription factor AP-1
LGP2: Laboratory of genetics and physiology 2
LPAIV: Low pathogenic avian influenza
LPS: Lipopolysaccharide
LRAT: Lecithin retinal acyltransferase
LRRK1: Leucine-rich repeat kinase 1
LY86: Lymphocyte antigen 86 gene
MAP3K8: mitogen-activated protein kinase kinase kinase 8
MCH: Melanin concentrating hormone
MDA5: Melanoma differentiation-associated gene 5
NDV: NEWCASTLE disease virus
OASL: 2′-5′-oligoadenylate synthase-like protein 1-like
PBMC: Peripheral blood mononuclear cells
PCA: Principal component analysis
PCK1: Phosphoenolpyruvate carboxykinase 1
PMCH: Pro-melanin-concentrating hormone
PMN: Polymorphonuclear leukocytes
RBCs: Red blood cells
RBP4: Retinol binding protein 4
RIG-I: Retinoic acid-inducible gene I
RNA: Ribonucleic acid
RNA-seq: RNA sequencing
RSAD2: Radical S-adenosyl methionine domain containing 2
RT-PCR: Reverse transcription polymerase chain reaction
SAAL1: Serum amyloid A like 1
SARS: Severe acute respiratory syndrome
SERPINC1: Serpin family C member 1
SOCS3: Suppressor of cytokine signaling 3
SPACIA1: Synoviocyte proliferation-associated in collagen-induced arthritis 1
SPINK4: Serine peptidase inhibitor, kazal type 4
TAT: Tyrosine aminotransferase
TLR3: Toll-like receptor 3
TNF-α: Tumor necrosis factor-alpha
TYH: Tyrosine hydroxylase
WBCs: White blood cells
WNV: West Nile virus
