# Supplementary material for: Transcriptomic analysis of immune response to bacterial lipopolysaccharide in zebra finch (Taeniopygia guttata)
Source: BMC Genomics. 2019 Aug 14;20:647. doi: 10.1186/s12864-019-6016-3 (PMC6693190; doi:10.1186/s12864-019-6016-3)
Supplement: Supplementary file 7 — Gene ontology analyses for each tissue. Lists of 30 most significantly overrepresented GO terms for upregulated and downregulated genes of the hypothalamus, spleen, and RBCs. (DOCX 32 kb) [file 12864_2019_6016_MOESM7_ESM.docx]

Supplemental Information

**Supplemental Table 1**. List of 30 most significantly overrepresented Gene Ontology (GO) functional categories enriched (q ≤ 0.05) by the upregulated genes of the hypothalamus following lipopolysaccharide challenge. Columns show GO term descriptors, enrichment q-values, number of genes in our differentially regulated gene list that contribute to overrepresented GO categories, and total number of genes in the zebra finch genome annotated with GO terms from the category.

| GO Functional Category | *q*-value | Diff. genes | Total genes in category |
| --- | --- | --- | --- |
| Response to stress | 5.04E-06 | 92 | 1392 |
| Macromolecule localization | 5.04E-06 | 83 | 1195 |
| Cellular localization | 5.04E-06 | 80 | 1118 |
| Protein localization | 5.04E-06 | 74 | 1043 |
| Cellular protein localization | 5.04E-06 | 59 | 753 |
| Intracellular transport | 5.04E-06 | 54 | 653 |
| Protein catabolic process | 5.04E-06 | 40 | 410 |
| Cellular macromolecule localization | 5.34E-06 | 59 | 757 |
| Organic substance transport | 1.70E-05 | 71 | 1022 |
| Macromolecule catabolic process | 1.70E-05 | 44 | 509 |
| Intracellular protein transport | 4.57E-05 | 40 | 458 |
| Protein exit from endoplasmic reticulum | 4.57E-05 | 8 | 20 |
| Organelle organization | 4.94E-05 | 99 | 1659 |
| Cellular response to stress | 5.08E-05 | 59 | 824 |
| Catabolic process | 5.08E-05 | 58 | 806 |
| Establishment of protein localization | 5.08E-05 | 57 | 787 |
| Organic substance catabolic process | 5.08E-05 | 57 | 788 |
| Protein transport | 5.08E-05 | 52 | 689 |
| Proteolysis involved in cellular protein catabolic process | 5.08E-05 | 31 | 315 |
| Cellular protein catabolic process | 6.99E-05 | 32 | 337 |
| Retrograde protein transport, ER to cytosol | 0.000115907 | 7 | 17 |
| Endoplasmic reticulum to cytosol transport | 0.000115907 | 7 | 17 |
| Mitochondrion organization | 0.000116611 | 29 | 297 |
| Cellular macromolecule catabolic process | 0.000121994 | 36 | 418 |
| Negative regulation of response to stimulus | 0.000128254 | 48 | 642 |
| Response to endoplasmic reticulum stress | 0.000138692 | 16 | 108 |
| Establishment of localization in cell | 0.000148164 | 58 | 845 |
| Cellular response to chemical stimulus | 0.000344313 | 67 | 1057 |
| Regulation of localization | 0.000392033 | 69 | 1105 |
| Proteasomal protein catabolic process | 0.000392033 | 19 | 160 |

**Supplemental Table 2.** List of 30 most significantly overrepresented Gene Ontology (GO) functional categories enriched (q ≤ 0.05) by the downregulated genes of the hypothalamus following lipopolysaccharide challenge. Columns show GO term descriptors, enrichment q-values, number of genes in our differentially regulated gene list that contribute to overrepresented GO categories, and total number of genes in the zebra finch genome annotated with GO terms from the category.

| GO Functional Category | *q*-value | Diff. genes | Total genes in category |
| --- | --- | --- | --- |
| Small molecule metabolic process | 5.26E-06 | 25 | 880 |
| Regulation of hormone levels | 5.26E-06 | 13 | 211 |
| Feeding behavior | 0.00012138 | 6 | 38 |
| Response to chemical | 0.00014978 | 32 | 1653 |
| Cell-cell signaling | 0.00017307 | 17 | 554 |
| Hormone transport | 0.00017307 | 9 | 137 |
| Hormone metabolic process | 0.000468136 | 7 | 84 |
| Small molecule biosynthetic process | 0.000690636 | 10 | 215 |
| Hormone secretion | 0.00070793 | 8 | 130 |
| Regulation of biological quality | 0.00086916 | 28 | 1587 |
| Organonitrogen compound metabolic process | 0.00086916 | 21 | 994 |
| Single-organism biosynthetic process | 0.00086916 | 16 | 595 |
| Response to oxygen-containing compound | 0.00086916 | 14 | 482 |
| Behavior | 0.00086916 | 11 | 290 |
| Nitrogen compound transport | 0.00086916 | 11 | 287 |
| Signal release | 0.00086916 | 9 | 184 |
| Organic hydroxy compound metabolic process | 0.00086916 | 8 | 147 |
| Glial cell differentiation | 0.00086916 | 7 | 100 |
| Cellular hormone metabolic process | 0.00086916 | 5 | 44 |
| Positive regulation of nucleotide metabolic process | 0.00086916 | 5 | 42 |
| Positive regulation of hormone secretion | 0.00086916 | 5 | 43 |
| Oligodendrocyte differentiation | 0.00086916 | 5 | 42 |
| Positive regulation of purine nucleotide metabolic process | 0.00086916 | 5 | 42 |
| Regulation of purine nucleotide metabolic process | 0.001098616 | 6 | 78 |
| Regulation of nucleotide metabolic process | 0.001215574 | 6 | 81 |
| Positive regulation of cAMP metabolic process | 0.001215574 | 4 | 25 |
| Positive regulation of cAMP biosynthetic process | 0.001215574 | 4 | 25 |
| Gliogenesis | 0.001290233 | 7 | 121 |
| Response to nitrogen compound | 0.001494132 | 10 | 273 |
| Gluconeogenesis | 0.001739481 | 4 | 28 |

**Supplemental Table 3**. List of 30 most significantly overrepresented Gene Ontology (GO) functional categories enriched (q ≤ 0.05) by the upregulated genes of the spleen following lipopolysaccharide challenge. Columns show GO term descriptors, enrichment q-values, number of genes in our differentially regulated gene list that contribute to overrepresented GO categories, and total number of genes in the zebra finch genome annotated with GO terms from the category.

| GO Functional Category | *q*-value | Diff. genes | Total genes in category |
| --- | --- | --- | --- |
| Immune system process | 2.93E-14 | 71 | 916 |
| Response to external biotic stimulus | 3.40E-13 | 35 | 260 |
| Response to other organism | 3.40E-13 | 35 | 260 |
| Response to biotic stimulus | 1.48E-12 | 35 | 275 |
| Response to external stimulus | 1.63E-12 | 61 | 788 |
| Immune response | 5.87E-12 | 42 | 416 |
| Response to stress | 7.70E-12 | 84 | 1392 |
| Defense response | 7.70E-12 | 42 | 422 |
| Regulation of immune system process | 2.41E-10 | 43 | 490 |
| Regulation of response to stimulus | 1.04E-09 | 86 | 1585 |
| Response to lipopolysaccharide | 1.21E-09 | 17 | 77 |
| Cell death | 2.01E-09 | 55 | 801 |
| Positive regulation of response to stimulus | 2.01E-09 | 55 | 800 |
| Response to molecule of bacterial origin | 2.80E-09 | 17 | 82 |
| Regulation of response to stress | 4.22E-09 | 42 | 521 |
| Negative regulation of cellular process | 6.07E-09 | 97 | 1973 |
| Regulation of cell death | 9.53E-09 | 47 | 651 |
| Positive regulation of immune system process | 9.53E-09 | 30 | 294 |
| Cytokine production | 9.53E-09 | 27 | 239 |
| Response to bacterium | 1.32E-08 | 19 | 118 |
| Cellular response to chemical stimulus | 1.34E-08 | 63 | 1057 |
| Cell activation | 1.34E-08 | 32 | 338 |
| Multi-organism process | 1.44E-08 | 45 | 617 |
| Programmed cell death | 2.90E-08 | 50 | 750 |
| Regulation of intracellular signal transduction | 4.01E-08 | 50 | 758 |
| Regulation of signaling | 5.20E-08 | 74 | 1398 |
| Regulation of cytokine production | 5.55E-08 | 24 | 210 |
| Regulation of programmed cell death | 1.04E-07 | 43 | 613 |
| Response to organic substance | 1.26E-07 | 60 | 1045 |
| Regulation of cell communication | 1.50E-07 | 72 | 1380 |

**Supplemental Table 4**. List of 30 most significantly overrepresented Gene Ontology (GO) functional categories enriched (q ≤ 0.05) by the downregulated genes of the spleen following lipopolysaccharide challenge. Columns show GO term descriptors, enrichment q-values, number of genes in our differentially regulated gene list that contribute to overrepresented GO categories, and total number of genes in the zebra finch genome annotated with GO terms from the category.

| GO Functional Category | *q*-value | Diff. genes | Total genes in category |
| --- | --- | --- | --- |
| Small molecule metabolic process | 1.44E-42 | 118 | 880 |
| Organic acid metabolic process | 1.03E-31 | 72 | 423 |
| Carboxylic acid metabolic process | 1.03E-31 | 69 | 382 |
| Oxoacid metabolic process | 1.03E-31 | 69 | 384 |
| Oxidation-reduction process | 1.45E-24 | 78 | 639 |
| Small molecule catabolic process | 3.75E-24 | 35 | 111 |
| Single-organism catabolic process | 2.89E-22 | 54 | 335 |
| Monocarboxylic acid metabolic process | 4.87E-21 | 43 | 218 |
| Organonitrogen compound catabolic process | 2.24E-19 | 31 | 113 |
| Alpha-amino acid metabolic process | 7.56E-19 | 29 | 100 |
| Organic acid catabolic process | 1.11E-18 | 27 | 85 |
| Single-organism biosynthetic process | 1.65E-18 | 66 | 595 |
| Small molecule biosynthetic process | 6.53E-17 | 38 | 215 |
| Organic acid biosynthetic process | 1.44E-16 | 30 | 130 |
| Alpha-amino acid catabolic process | 8.19E-16 | 16 | 28 |
| Lipid metabolic process | 3.68E-15 | 60 | 585 |
| Cellular lipid metabolic process | 1.56E-14 | 49 | 417 |
| Carboxylic acid biosynthetic process | 1.56E-14 | 27 | 121 |
| Organonitrogen compound metabolic process | 6.13E-14 | 79 | 994 |
| Fatty acid metabolic process | 1.76E-13 | 27 | 133 |
| Carboxylic acid catabolic process | 3.26E-13 | 21 | 76 |
| Cellular amino acid catabolic process | 5.63E-13 | 15 | 33 |
| Catabolic process | 4.26E-12 | 66 | 806 |
| Dicarboxylic acid metabolic process | 1.06E-11 | 15 | 39 |
| Cellular amino acid metabolic process | 2.07E-11 | 25 | 137 |
| Organic substance catabolic process | 1.38E-10 | 62 | 788 |
| Aromatic amino acid family metabolic process | 5.46E-10 | 11 | 22 |
| Cellular catabolic process | 7.92E-10 | 53 | 638 |
| Alcohol metabolic process | 1.40E-09 | 19 | 92 |
| Lipid homeostasis | 4.53E-09 | 13 | 40 |

**Supplemental Table 5**. List of 30 most significantly overrepresented Gene Ontology (GO) functional categories enriched (q ≤ 0.05) by the upregulated genes of the red blood cells following lipopolysaccharide challenge. Columns show GO term descriptors, enrichment q-values, number of genes in our differentially regulated gene list that contribute to overrepresented GO categories, and total number of genes in the zebra finch genome annotated with GO terms from the category.

| GO Functional Category | *q*-value | Diff. genes | Total genes in category |
| --- | --- | --- | --- |
| Immune system process | 1.43E-05 | 24 | 916 |
| Immune system development | 1.46E-05 | 16 | 423 |
| Hemopoiesis | 1.46E-05 | 15 | 372 |
| Hematopoietic or lymphoid organ development | 2.82E-05 | 15 | 400 |
| Leukocyte differentiation | 8.52E-05 | 11 | 223 |
| Cytokine production | 0.000140984 | 11 | 239 |
| Immune response | 0.000160706 | 14 | 416 |
| Lymphocyte activation | 0.000199514 | 11 | 255 |
| Regulation of cytokine production | 0.000223344 | 10 | 210 |
| Cellular nitrogen compound catabolic process | 0.000241921 | 8 | 126 |
| Heterocycle catabolic process | 0.000241921 | 8 | 126 |
| Regulation of cytokine biosynthetic process | 0.00024865 | 5 | 33 |
| Leukocyte activation | 0.000311795 | 11 | 287 |
| Innate immune response | 0.000311795 | 8 | 135 |
| Organic cyclic compound catabolic process | 0.000311795 | 8 | 136 |
| Cytokine biosynthetic process | 0.000311795 | 5 | 36 |
| Cytokine metabolic process | 0.000315313 | 5 | 37 |
| Cellular response to interferon-gamma | 0.000345965 | 4 | 18 |
| Regulation of immune system process | 0.000394806 | 14 | 490 |
| Response to stress | 0.00040824 | 25 | 1392 |
| Response to interferon-gamma | 0.000693871 | 4 | 22 |
| Cellular response to mechanical stimulus | 0.000771638 | 4 | 23 |
| Interferon-alpha production | 0.000771638 | 3 | 8 |
| Cell differentiation | 0.000889564 | 27 | 1669 |
| Positive regulation of macromolecule metabolic process | 0.000889564 | 23 | 1320 |
| Cell activation | 0.000889564 | 11 | 338 |
| Myeloid cell differentiation | 0.000889564 | 8 | 178 |
| Cytokine-mediated signaling pathway | 0.000889564 | 7 | 129 |
| Aromatic compound catabolic process | 0.000889564 | 7 | 126 |
| Type I interferon production | 0.000889564 | 4 | 27 |

**Supplemental Table 6.** List of most significantly overrepresented Gene Ontology (GO) functional categories enriched (q ≤ 0.05) by the downregulated genes of the red blood cells following lipopolysaccharide challenge. Columns show GO term descriptors, enrichment q-values, number of genes in our differentially regulated gene list that contribute to overrepresented GO categories, and total numbers of genes of zebra finch annotated with GO terms of category.

| GO Functional Category | *q*-value | Diff. genes | Total genes in category |
| --- | --- | --- | --- |
| Regulation of cellular component organization | 0.02926416 | 12 | 1098 |
| Receptor metabolic process | 0.02926416 | 4 | 92 |
| Regeneration | 0.02926416 | 3 | 42 |
| Regulation of vesicle fusion | 0.02926416 | 2 | 7 |
| Positive regulation of vesicle fusion | 0.02926416 | 2 | 5 |
| Negative regulation of interleukin-8 production | 0.02926416 | 2 | 6 |
| Intermediate filament organization | 0.02926416 | 2 | 9 |
| Negative regulation of cellular component organization | 0.036804127 | 6 | 300 |
